# Supplementary material for: Hot carriers perspective on the nature of traps in perovskites
Source: Nat Commun. 2020 Jun 1;11:2712. doi: 10.1038/s41467-020-16463-7 (PMC7264280; doi:10.1038/s41467-020-16463-7)
Supplement: Supplementary file 1 — Supplementary Information [file 41467_2020_16463_MOESM1_ESM.pdf]

SUPPORTING INFORMATION

Hot Carriers Perspective on the Nature of Traps in Perovskites

Marcello Righetto [†,1], *Swee Sien Lim*[†,1], *David Giovanni*[†,1], *Jia Wei Melvin Lim*[1], *Qiannan Zhang*[1], *Sankaran Ramesh* [1], *Eugene Yong Kang Tay* [1], *Tze Chien Sum*[1,*]

[1]Division of Physics and Applied Physics, School of Physical and Mathematical Sciences, Nanyang Technological University, 21 Nanyang Link, Singapore 637371, Singapore

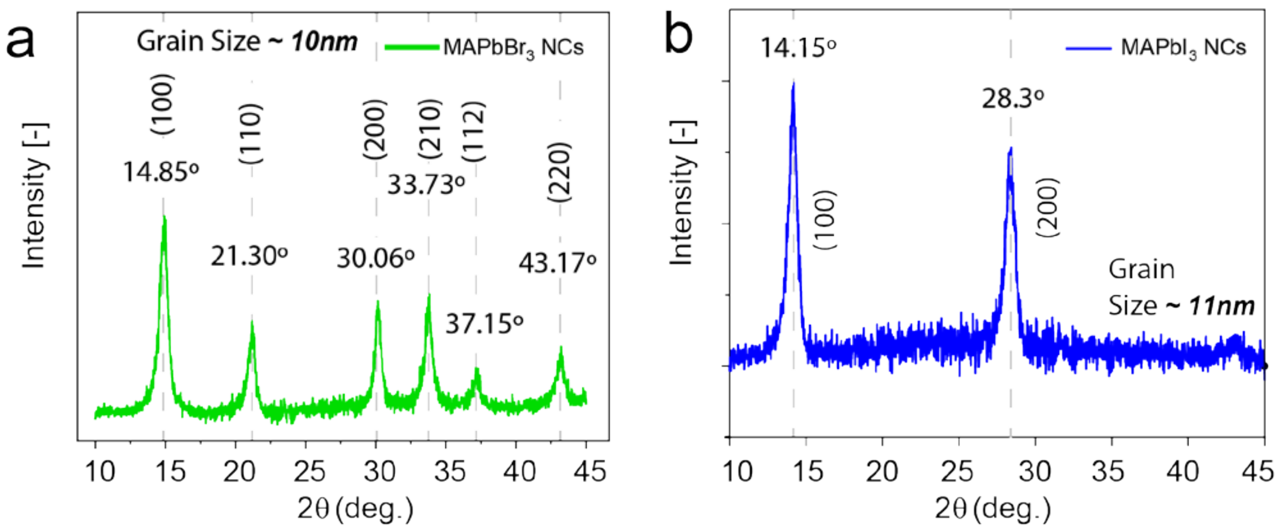

Supplementary Figure 1: X-Ray Characterization of Perovskite Nanocrystals. X-ray diffraction pattern of (a) $MAPbBr_3$ and (b) $MAPbI_3$ NCs, with grain size rough estimate from Debye Scherrer analysis). Both $MAPbBr_3$ and $MAPbI_3$ NCs have a cubic structure ($Pm\bar{3}m$ space group). Lines represent the XRD intensity.

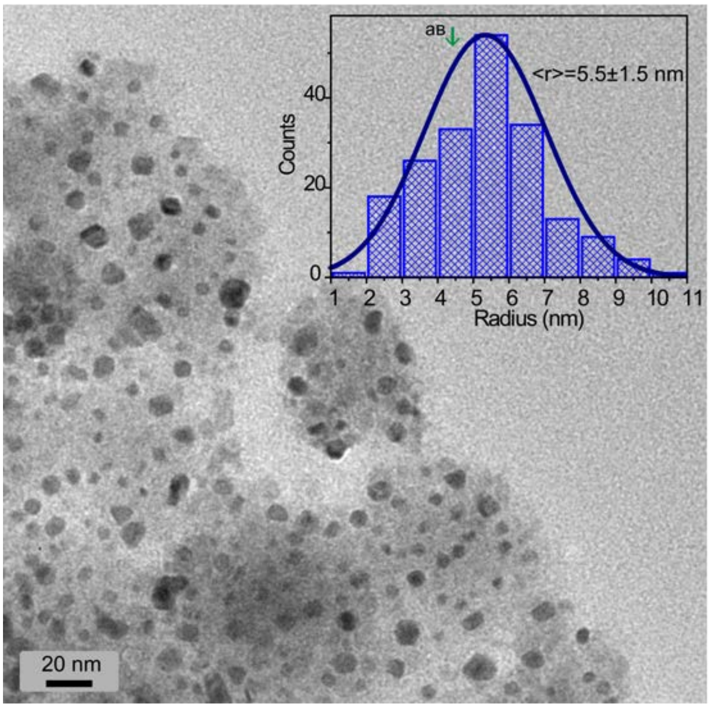

Supplementary Figure 2 : **Electron Microscopy Characterization of $MAPbI_3$ nanocrystals.** TEM micrograph of $MAPbI_3$ NCs. Scale bar is 20 nm. Inset: Size distribution analysis with line representing the gaussian fit of the size distribution.

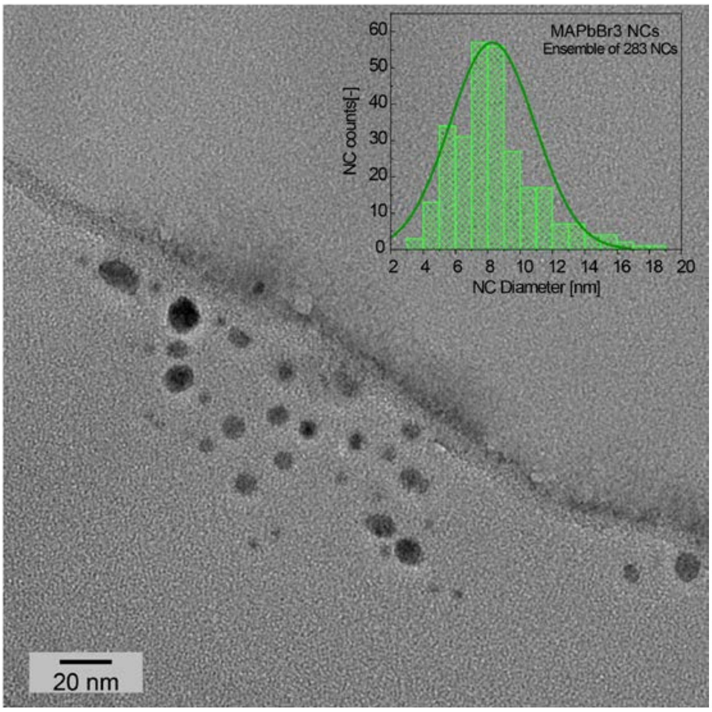

Supplementary Figure 3: **Electron Microscopy Characterization of $MAPbBr_3$ nanocrystals.** TEM micrograph of $MAPbBr_3$ NCs. Scale bar is 20 nm. Inset: Size distribution analysis with line representing the gaussian fit of the size distribution.

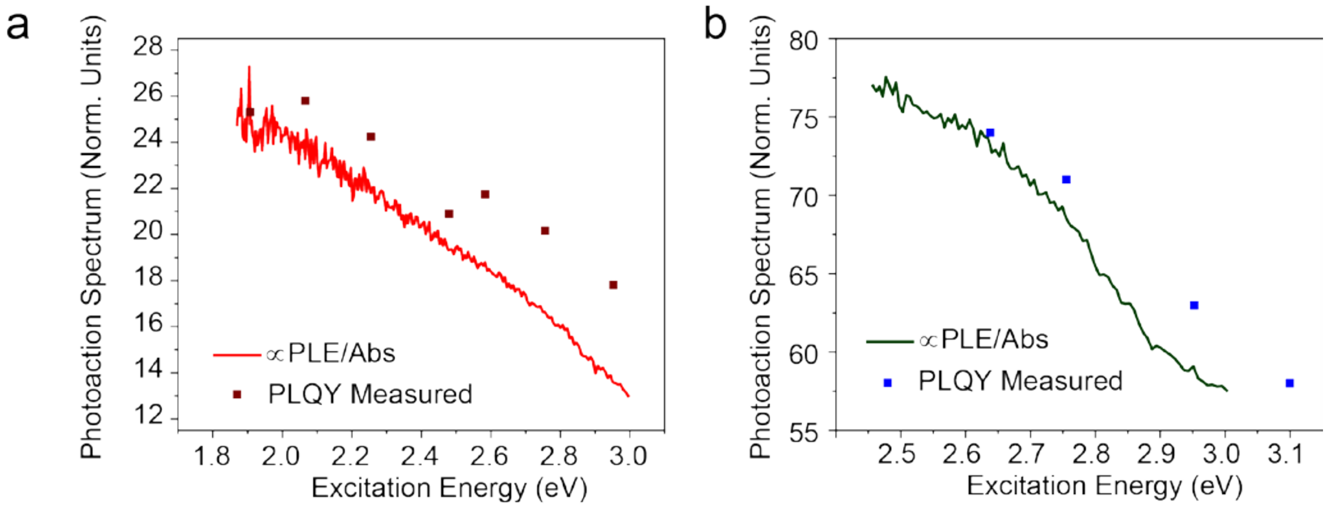

Supplementary Figure 4: **Photoaction spectra in iodide and bromide perovskite nanocrystals.** Comparison between photoaction spectra for $MAPbI_3$ (a) and $MAPbBr_3$ (b) colloidal NCs. Solid lines represent the normalized photoaction spectrum obtained as ratio between photoluminescence excitation and absorption spectra. Wine and Blue squares represent the PLQY directly measured.

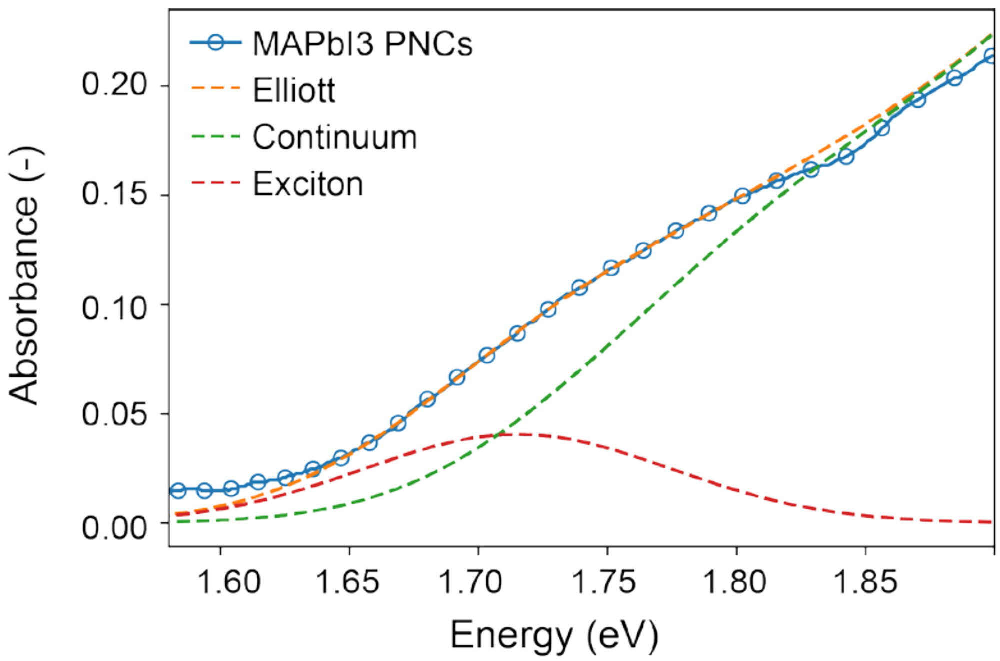

Supplementary Figure 5: **Elliott Fit for methylammonium lead iodide nanocrystals absorption spectrum.** Fitting of the absorption spectrum of $MAPbI_3$ NCs with the Elliott formula, deconvolving the contributions due to free carrier absorption continuum (green), and excitonic (red).

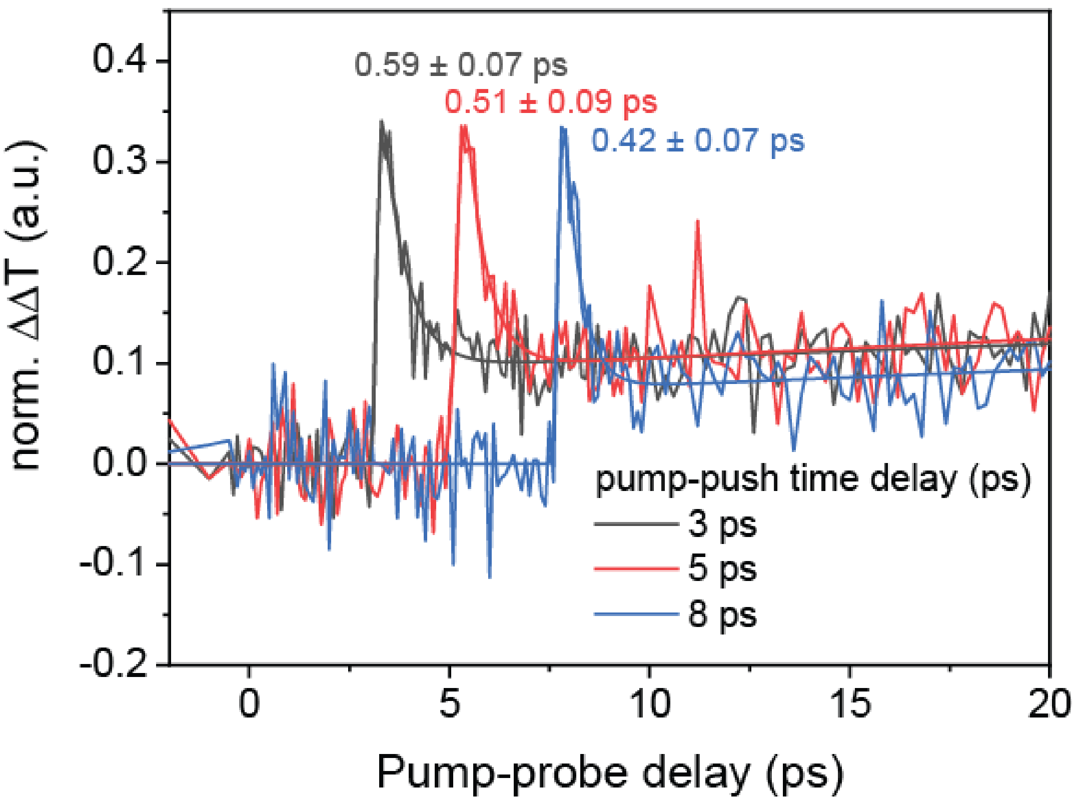

Supplementary Figure 6: Push delay dependence in pump-push-probe measurements. Normalized push delay-dependence kinetics for $MAPbI_3$ NC at pumped at 1.91 eV (10 µJ cm^{-2}, <N> = 0.8), probed at 1.7 eV, pushed at 1.03 eV (1 mJ cm^{-2}) with varying pump-push delays of 3 ps, 5 ps, and 8 ps. Thin lines prepresent the experimental data, thick lines represent the model fit.

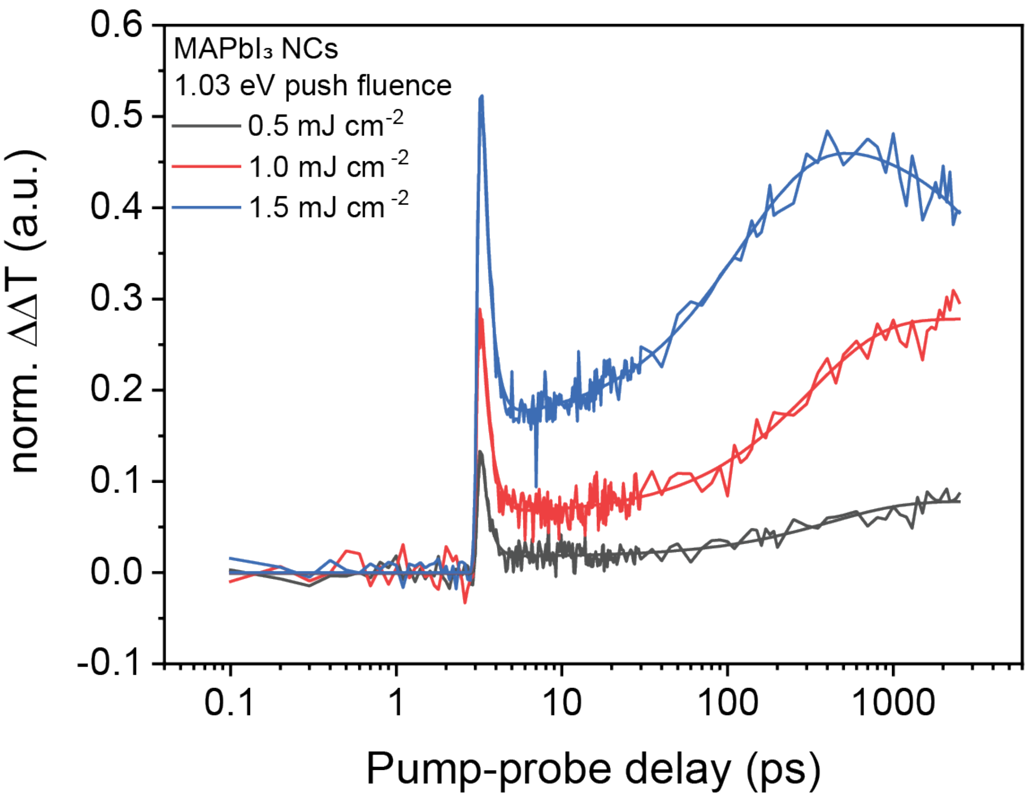

Supplementary Figure 7: Push fluence dependence in pump-push-probe measurements. Normalized pump-push-probe differential kinetics of pristine $MAPbI_3$ NCs in anhydrous toluene solutions. The samples were pumped at 2.07 eV (10 µJ cm^{-2}, <*N*> = 0.88), probed at 1.7 eV (in correspondence to the main bleaching peak), and with a push energy of 1.03 eV (0.5 to 1.5 mJ cm^{-2}). Thin lines prepresent the experimental data, thick lines represent the model fit.

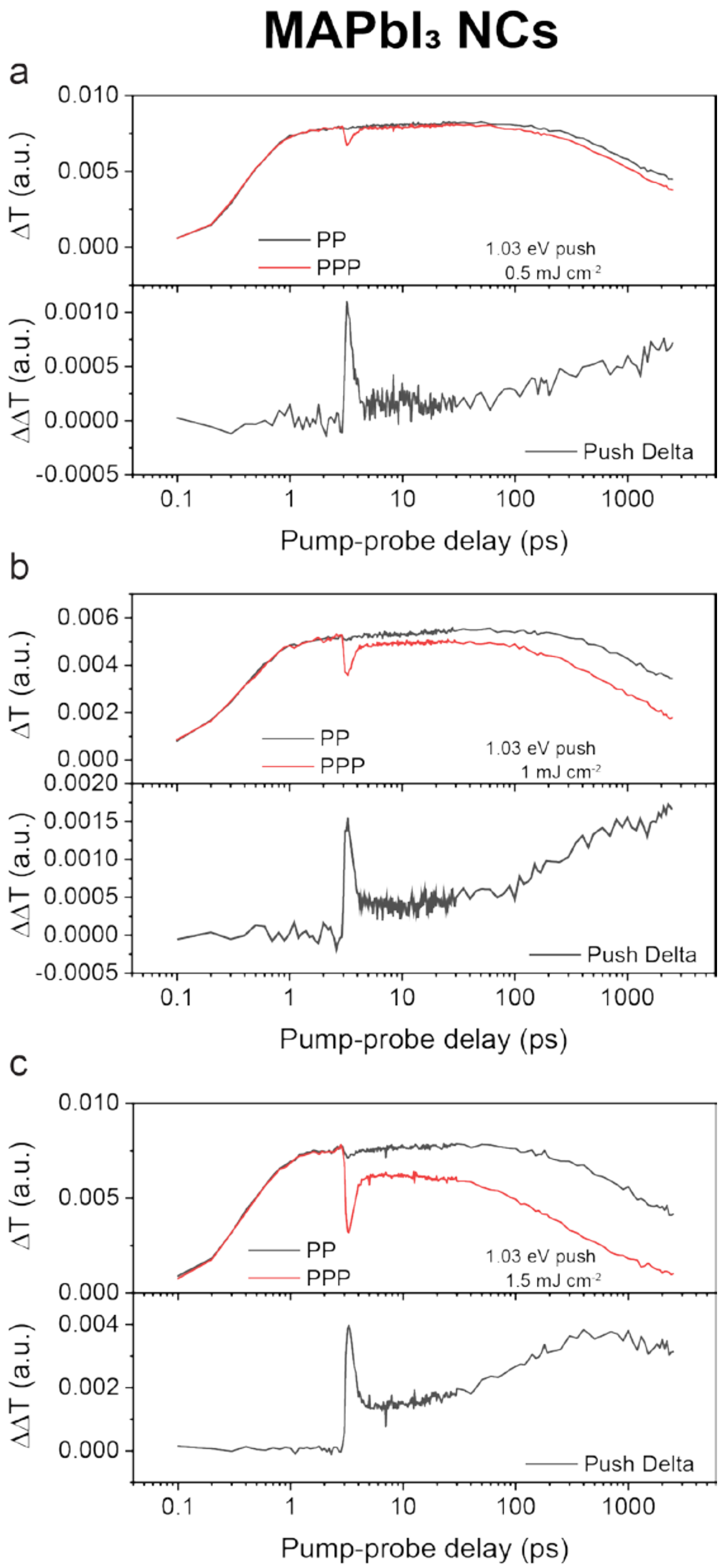

Supplementary Figure 8: **Normalized pump-push-probe data for $MAPbI_3$ nanocrystals.** Normalized pump-probe (black), pump-push-probe (red) and differential pump-push-probe (grey) kinetics for $MAPbI_3$ NCs in anhydrous toluene solutions. The samples were pumped at 2.07 eV (10 µJ cm^{-2}, <*N*> = 0.88), probed at 1.7 eV, and with a push energy of 1.03 eV (0.5 to 1.5 mJ cm^{-2}).

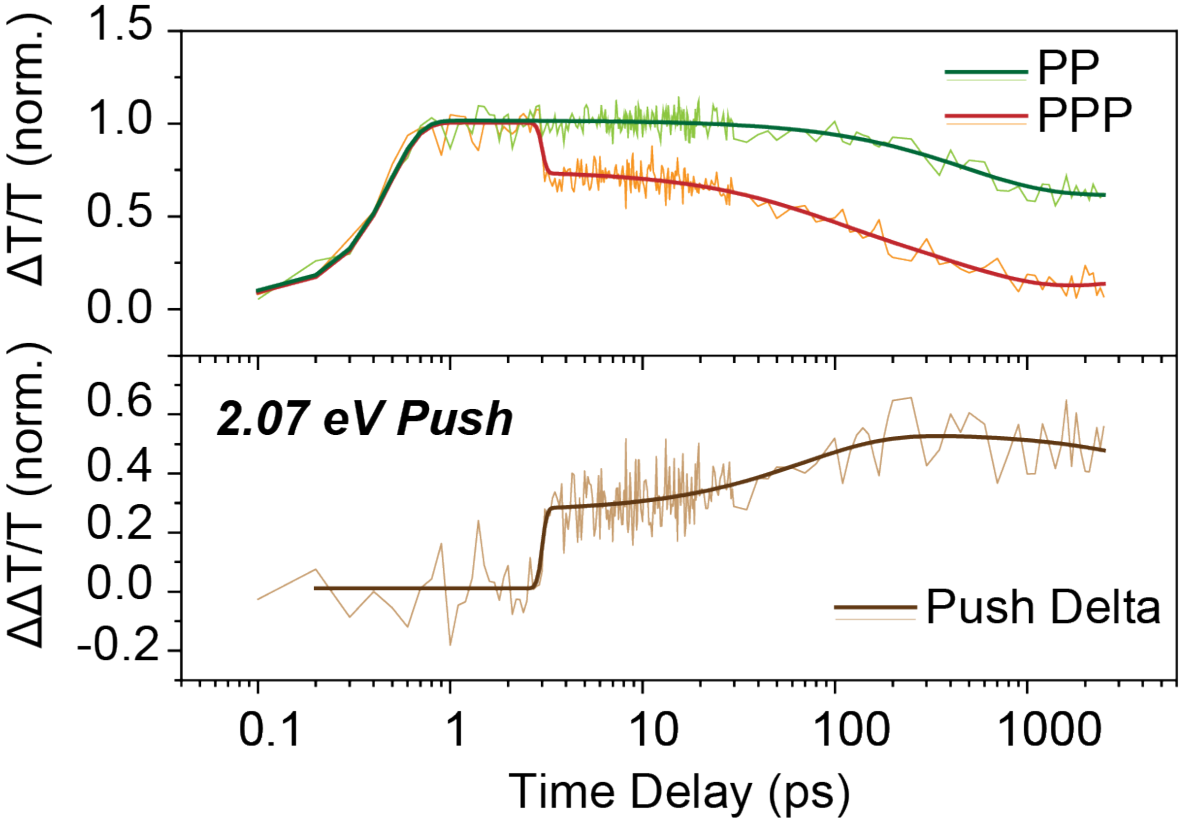

Supplementary Figure 9: **Visible push experiment in pump-push-probe for MAPbI$_3$ nanocrystals.** Pump-probe (PP) and pump-push-probe (PPP) kinetics pumped at 1.91 eV (10 µJ cm^{-2}, <*N*> = 0.8) and probed at 1.70 eV, with push energy of 2.07 eV (60 µJ cm^{-2}). Thin lines represent the experimental data, and thick lines are exponential model fits to the data.

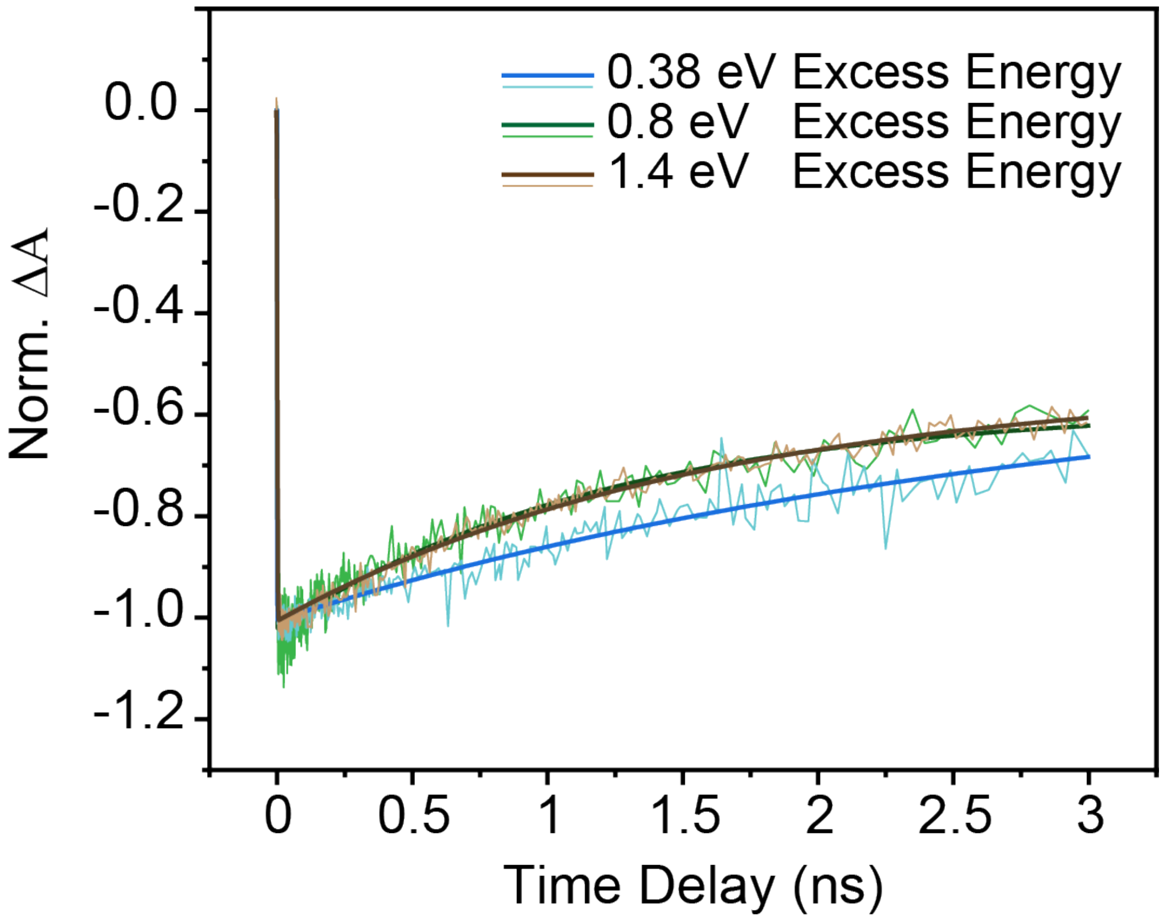

Supplementary Figure 10: **Pump-probe kinetics with different pump energies.** Excitation energy-resolved PP spectroscopy of $MAPbI_3$ NC. Pump energy was varied from 2.07 eV to 2.4 eV and 3.1 eV, while keeping constant the average N (<*N*> = 0.23), and probing at 1.7 eV. Thin lines represent the experimental data, and thick lines are exponential model fits to the data.

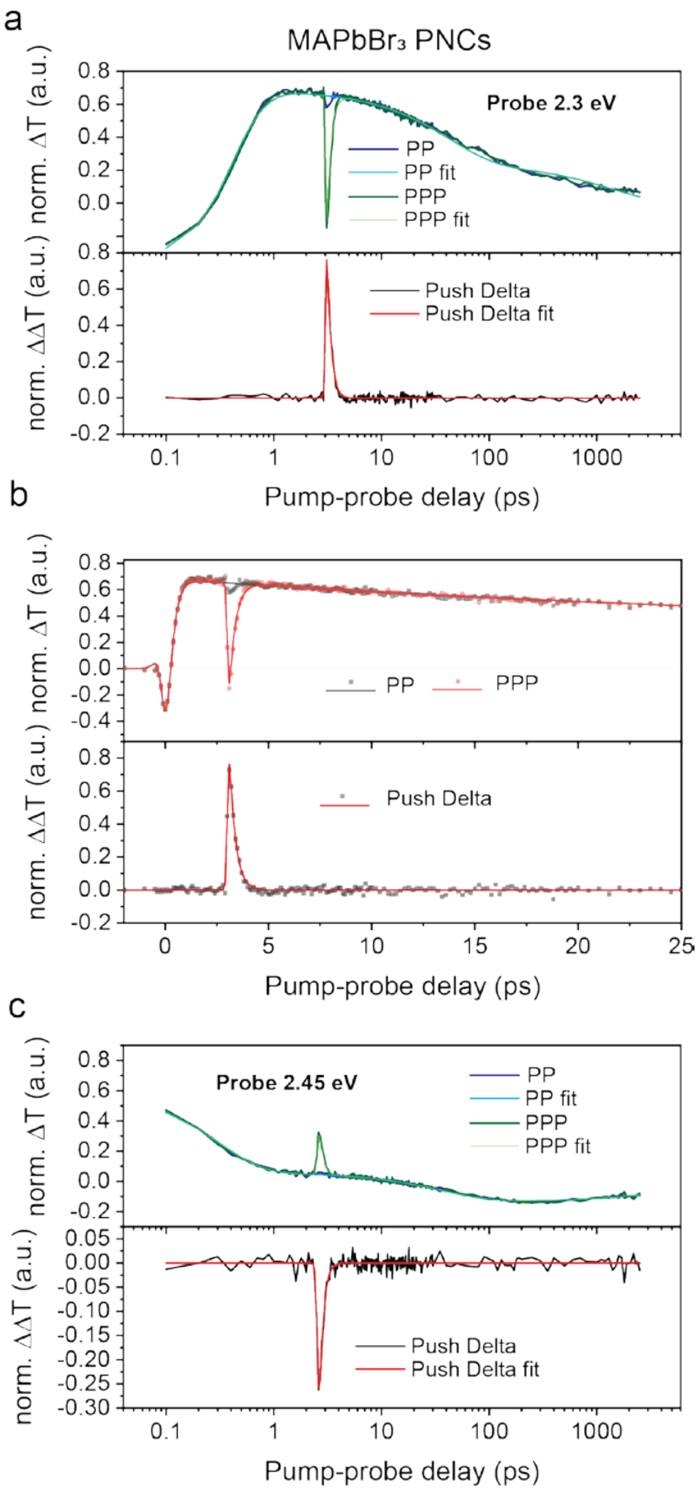

Supplementary Figure 11: Normalized pump-push-probe data for $MAPbBr_3$ nanocrystals. Normalized pump-push-probe kinetics for $MAPbBr_3$ NCs in anhydrous toluene solutions. The sample was pumped at 3.10 eV (40 µJ cm^{-2}), pushed at 1.03 eV (2.0 mJ cm^{-2}), and probed at 2.3 and 2.45 eV for the (a) and (c) panels, respectively. (b) Early time dynamics of normalized pump-push-probe kinetics for $MAPbBr_3$ NCs in anhydrous toluene solutions at 2.3 eV, revealing the presence of a PIA associated with the presence of hot carriers.

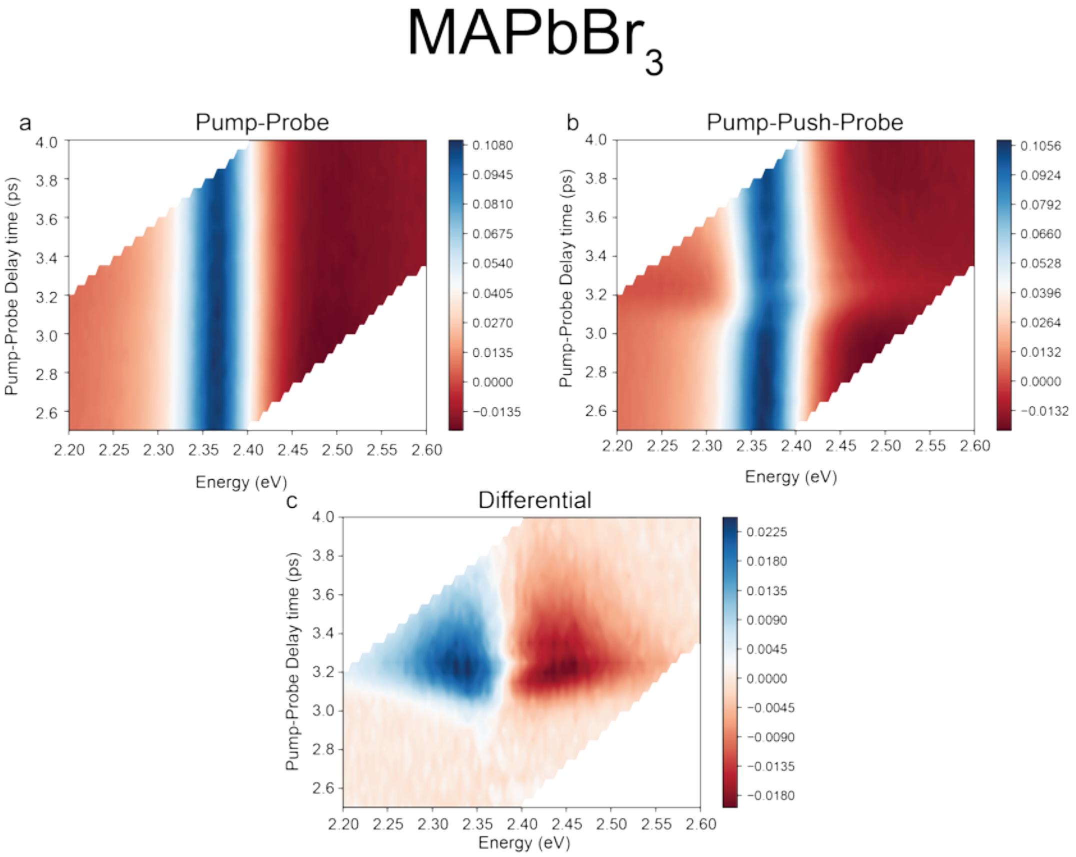

Supplementary Figure 12: **Pump-push-probe spectra for $MAPbBr_3$ nanocrystals.** Chirp-corrected pump-push-probe spectra of $MAPbBr_3$ NCs in anhydrous toluene solutions, pumped at 3.10 eV (40 μJ cm^{-2}), and pushed at 1.03 eV (2.0 mJ cm^{-2}).

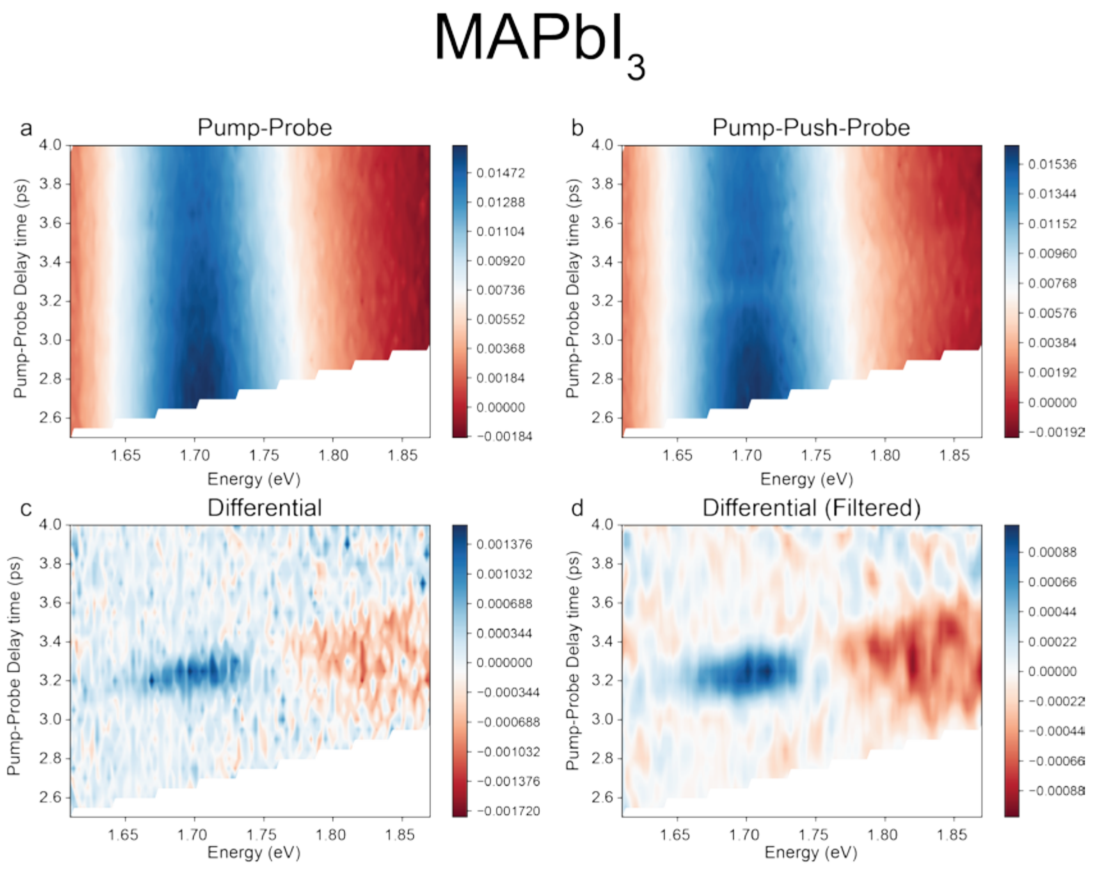

Supplementary Figure 13: **Pump-push-probe spectra for $MAPbI_3$ nanocrystals.** Chirp-corrected pump-push-probe spectra of $MAPbI_3$ NCs in anhydrous toluene, pumped at 2.07 eV (10 µJ cm^{-2}, $\langle N \rangle = 0.88$), and pushed at 1.03 eV (2.0 mJ cm^{-2}). A Savitzky-Golay filter was applied using a filter window length of 11 and a 5th-order polynomial to reduce the high-frequency noise for illustration purposes.

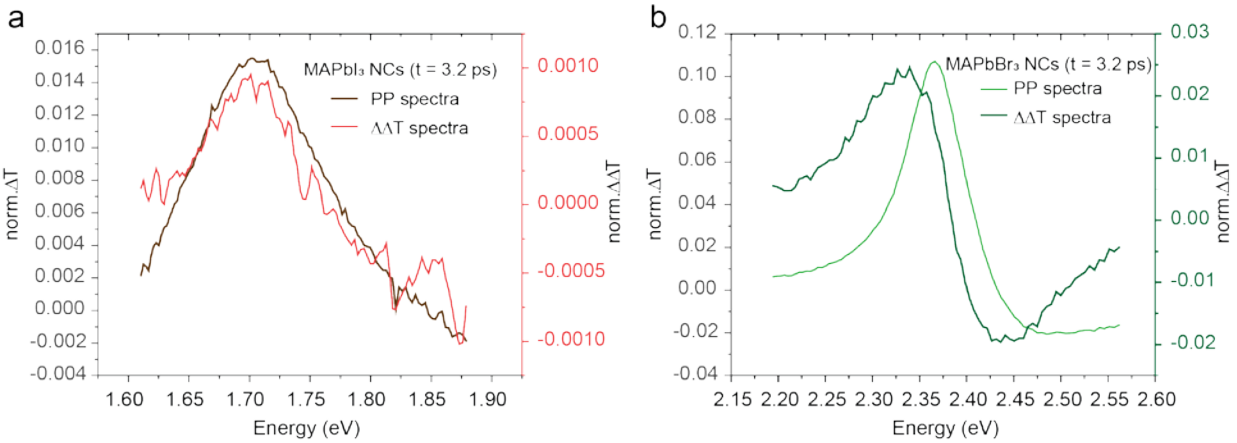

Supplementary Figure 14: Pump-push-probe spectra slices. Comparison of ΔΔT and PP spectra with 1.03 eV push for (a) $MAPbI_3$ and (b) $MAPbBr_3$ NCs from Supplementary 12 and 13, showing the different effects of the push on the two NCs systems. Panel (a): red line represents the ΔΔT and brown line represents the PP signal. Panel (b):dark green line represents the ΔΔT and light green line represents the PP signal.

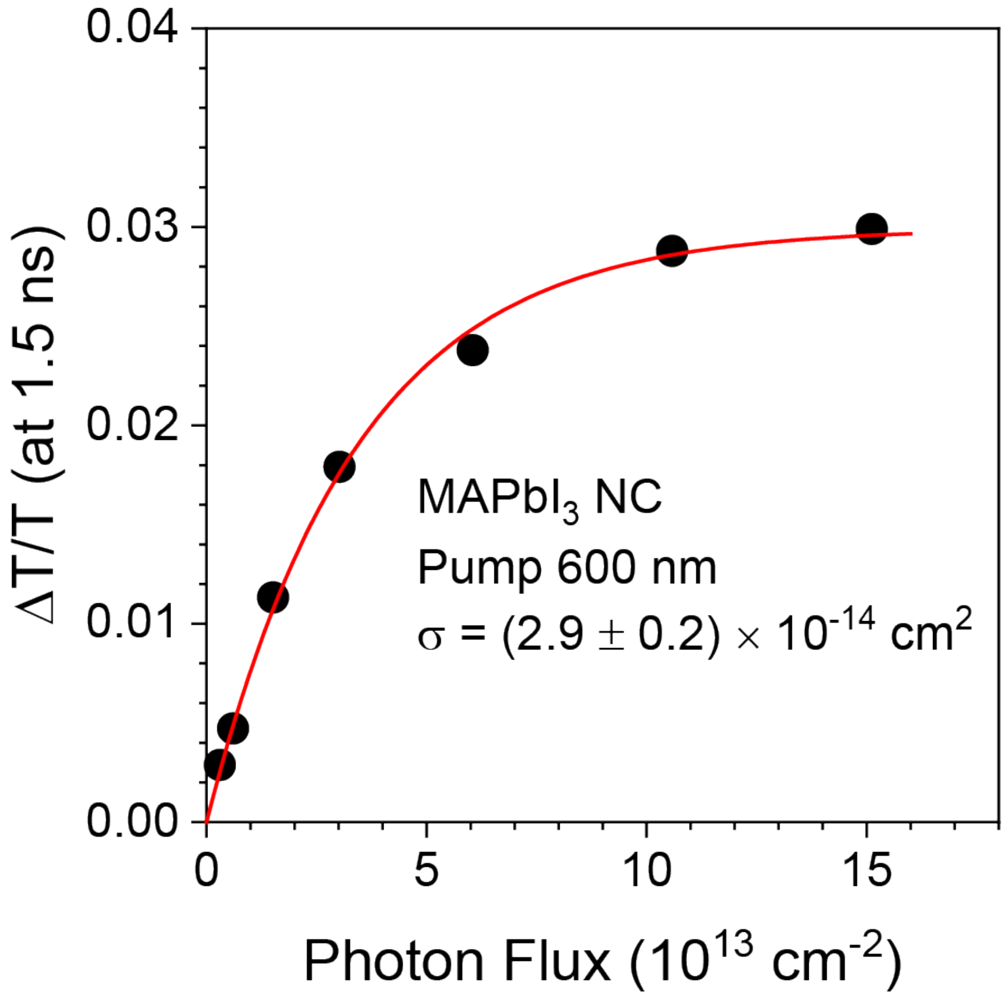

Supplementary Figure 15: **Saturation fit of cross section for $MAPbI_3$ nanocrystals.** Absorption cross-section calculation obtained from fluence-dependent PP (pumped at 3.1 eV, 600 nm). Black circles represent the experimental values, the red line represents the model fit.

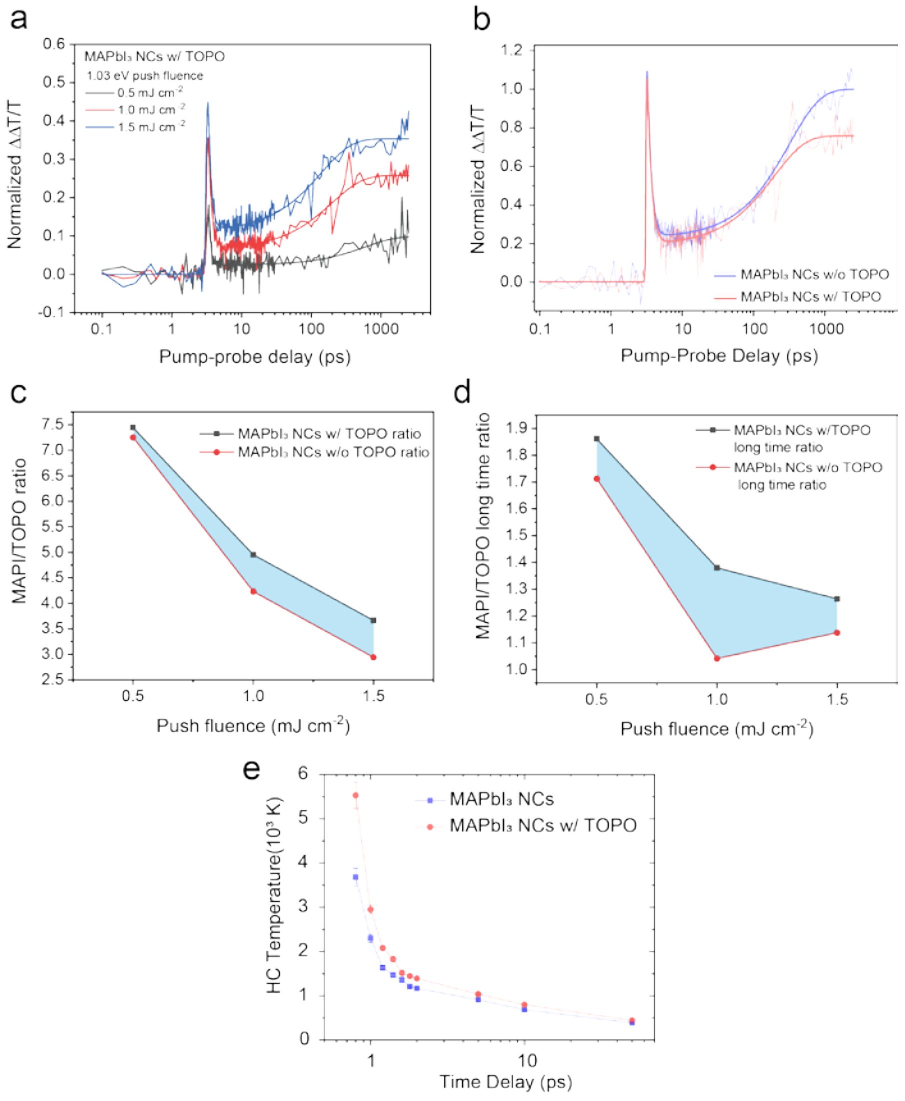

Supplementary Figure 16: **Comparision between pristine and ligand-exchanged nanocrystals.** (a) Normalized pump-push-probe differential kinetics of TOPO ligand exchanged $MAPbI_3$ NCs in anhydrous toluene solutions. The samples were pumped at 2.07 eV (10 µJ cm^{-2}, <*N*> = 0.88), probed at 1.7 eV (in correspondence to the main bleaching peak), and with a push energy of 1.03 eV (0.5 to 1.5 mJ cm^{-2}). (b) Comparison between differential pump-push-probe signals for pristine $MAPbI_3$ NCs (blue) and TOPO-ligand exchanged $MAPbI_3$ NCs (red). Samples were pumped at 2.07 eV (37 µJ cm^{-2}) and pushed with 1.03 eV pulses (1 mJ cm^{-2}). Raw data are represented by thin solid lines, while thick solid lines represent fits. (c),(d) Comparison of trapping efficiency between pristine and ligand exchanged $MAPbI_3$ NCs for short-time and long-time carrier losses. The distance between the black and red points (cyan area) represents the percentage of carrier preserved from trapping thanks to the additional TOPO passivation. (e) Comparison between hot carriers temperatures for pristine $MAPbI_3$ NCs (blue) and TOPO-ligand exchanged $MAPbI_3$ NCs (red). TA measurements were obtained using 3.1 eV (80 µJ cm^{-2}) and white light probe. HC temperatures (red circles for ligand-

exchanged and blue for pristine $MAPbI_3$ NCs) were extracted using the Boltzmann fit method. Lines are a guide for the eyes.

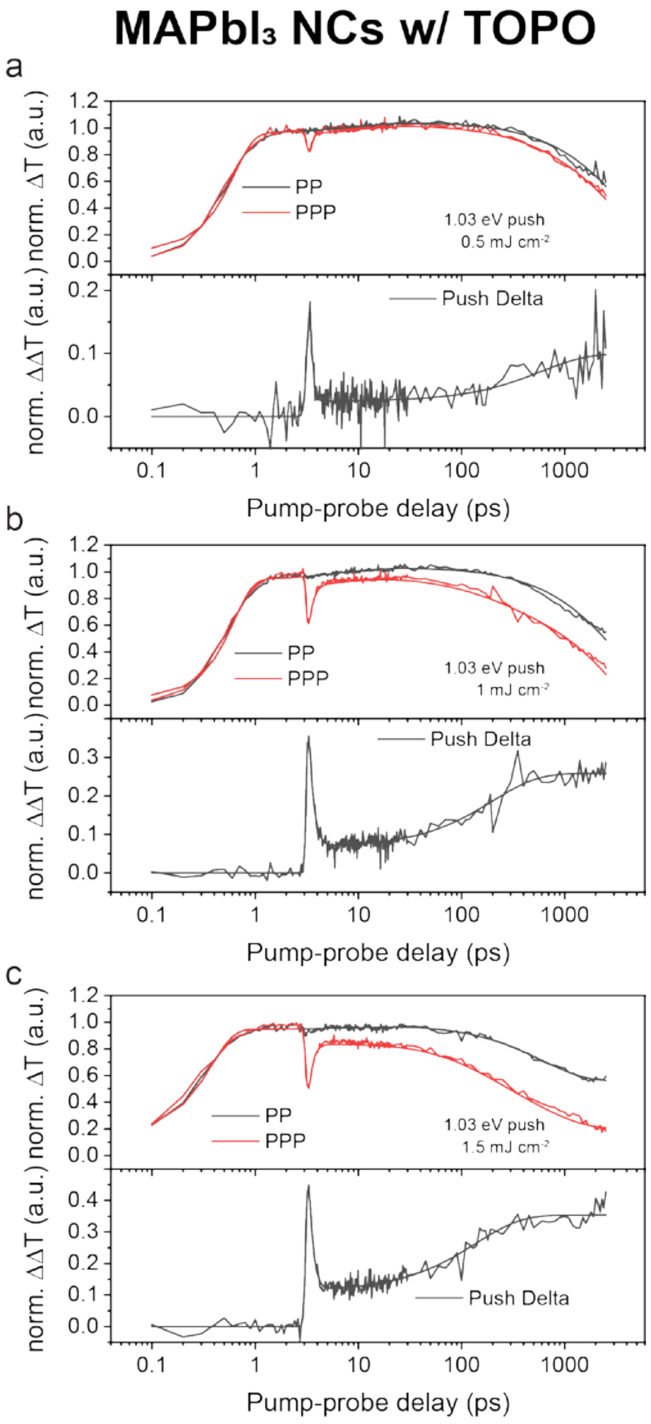

Supplementary Figure 17: **Normalized pump-push-probe data for ligand exchanged $MAPbI_3$ nanocrystals.** **N**ormalized pump-probe (black), pump-push-probe (red) and differential pump-push-probe (grey) kinetics for TOPO ligand exchanged $MAPbI_3$ NCs in anhydrous toluene solutions. The samples were pumped at 2.07 eV (10 µJ cm^{-2}, $\langle N \rangle = 0.88$), probed at 1.7 eV, and with a push energy of 1.03 eV (0.5 to 1.5 mJ cm^{-2}).

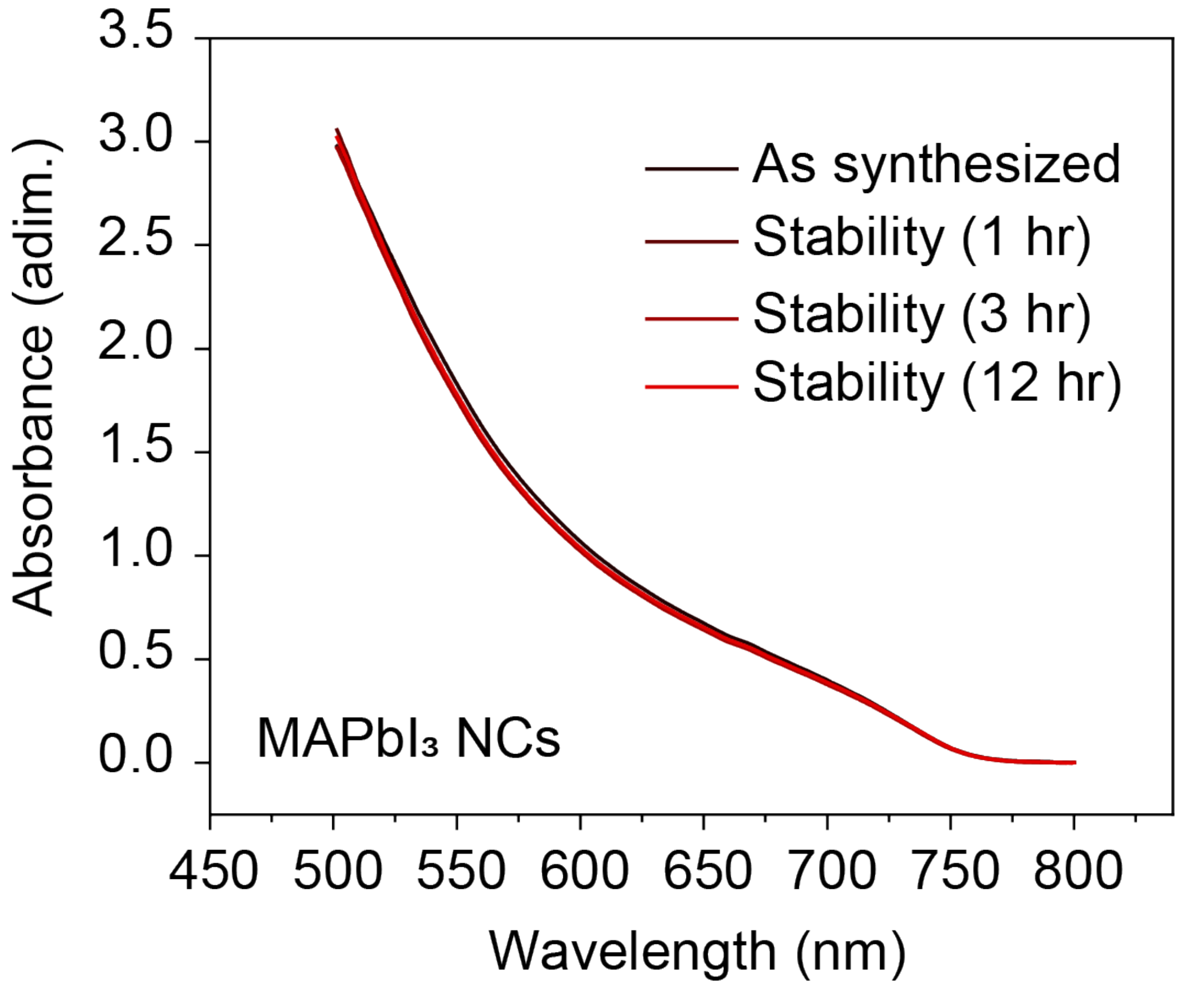

Supplementary Figure 18: Stability measurements for $MAPbI_3$ nanocrystals. Evolution of the absorption spectrum of $MAPbI_3$ colloidal NCs during repeated PPP experiments. Samples were stirred and excited with the following PPP scheme: pump 2.07 eV (at 10 μJ cm^{-2}), push 1.03 eV (at 1 mJ cm^{-2}). Lighter coloured lines correspond to longer times.

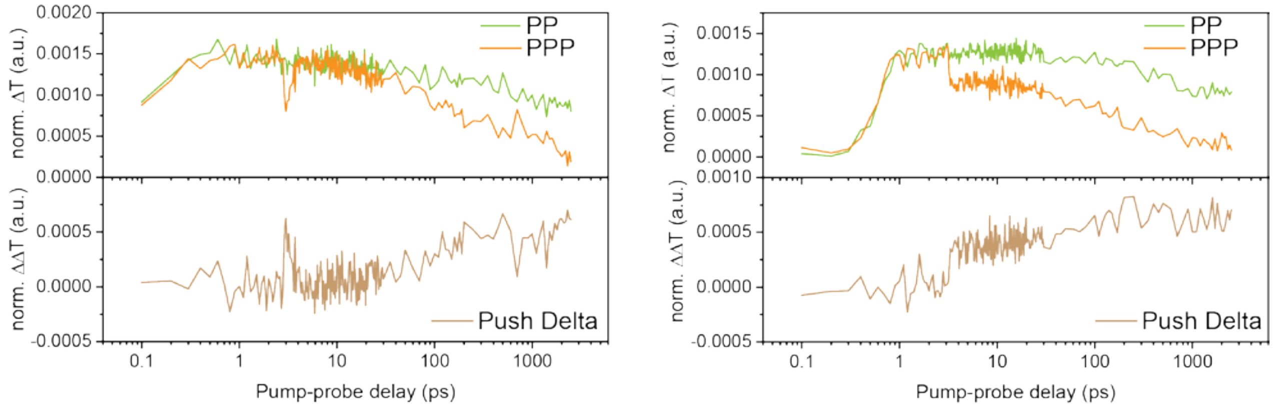

Supplementary Figure 19: **Raw pump-push-probe kinetics for $MAPbI_3$ nanocrystals.** Pump-push-probe differential spectrum of $MAPbI_3$ NCs in anhydrous toluene solutions, pumped at 2.07 eV (10 μJ cm^{-2}, $\langle N \rangle = 0.88$), probed at 1.70 eV, and pushed at 1.03 eV (1 mJ cm^{-2}). The positive ΔΔT signal (blue) implies a decrease in the PB signal, and a negative ΔΔT signal (red) an increase in the PB signal. (c-d) Pump-probe (PP) and pump-push-probe (PPP) kinetics pumped at 1.91 eV (10 μJ cm^{-2}, $\langle N \rangle = 0.8$) and probed at 1.70 eV, with push energy of (c) 1.03 eV (1 mJ cm^{-2}) and (d) 2.07 eV (60 μJ cm^{-2}). Green, orange, and brown lines correspond to PP, PPP, and push delta, respectively.

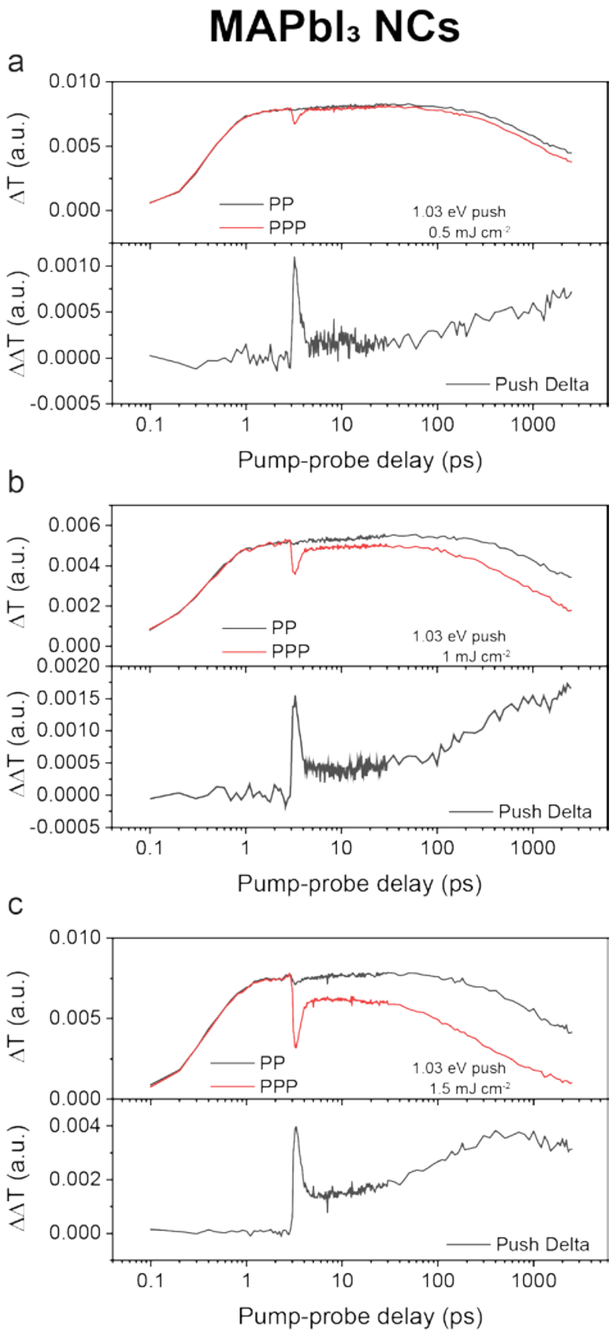

Supplementary Figure 20: **Raw pump-push-probe data for $MAPbI_3$ nanocrystals.** Pump-probe (black), pump-push-probe (red) and differential pump-push-probe (grey) kinetics for $MAPbI_3$ NCs in anhydrous toluene solutions. The samples were pumped at 2.07 eV (10 µJ cm^{-2}, <*N*> = 0.88), probed at 1.7 eV, and with a push energy of 1.03 eV (0.5 to 1.5 mJ cm^{-2}). Light grey, red, and dark grey correspond to PP, PPP, and push delta, respectively.

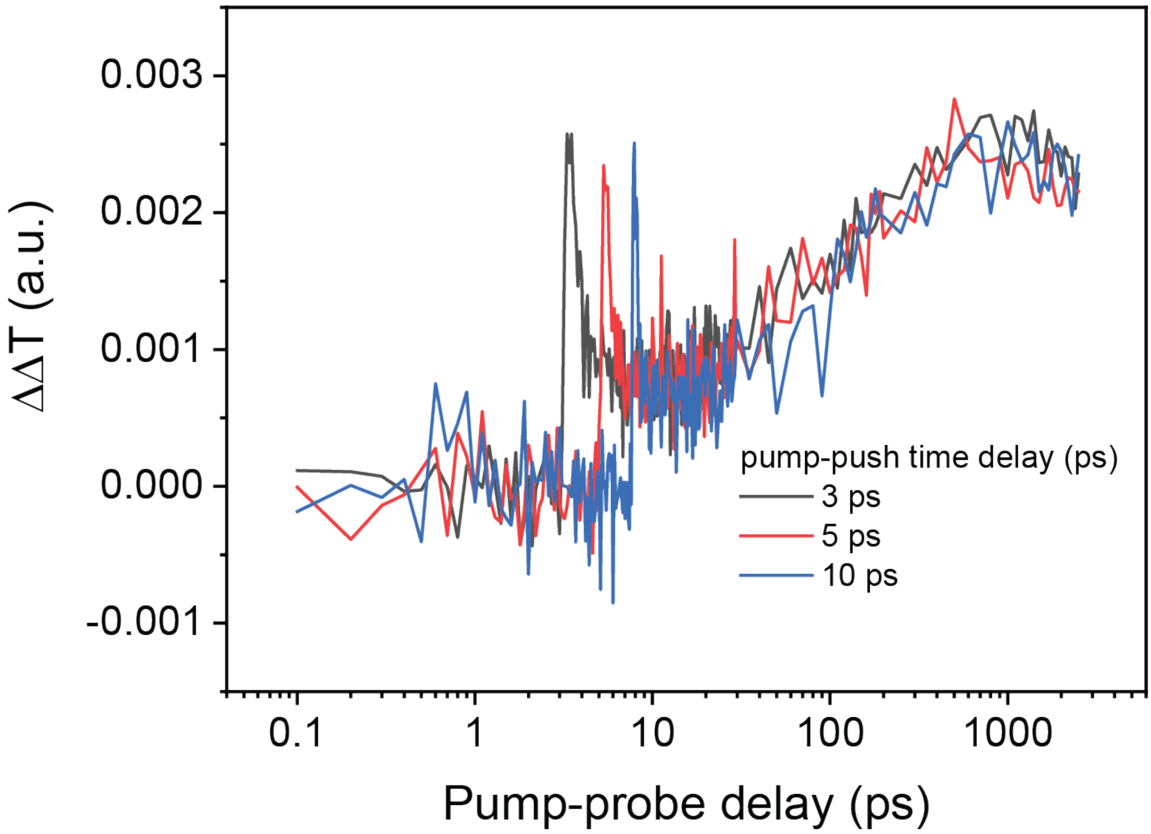

Supplementary Figure 21: **Raw push delay dependent pump-push-probe measurements.** Push delay-dependence kinetics for $MAPbI_3$ NC at pumped at 1.91 eV (10 µJ cm^{-2}, <*N*> = 0.8), probed at 1.7 eV, pushed at 1.03 eV (1 mJ cm^{-2}) with varying pump-push delays of 3 ps, 5 ps, and 10 ps.

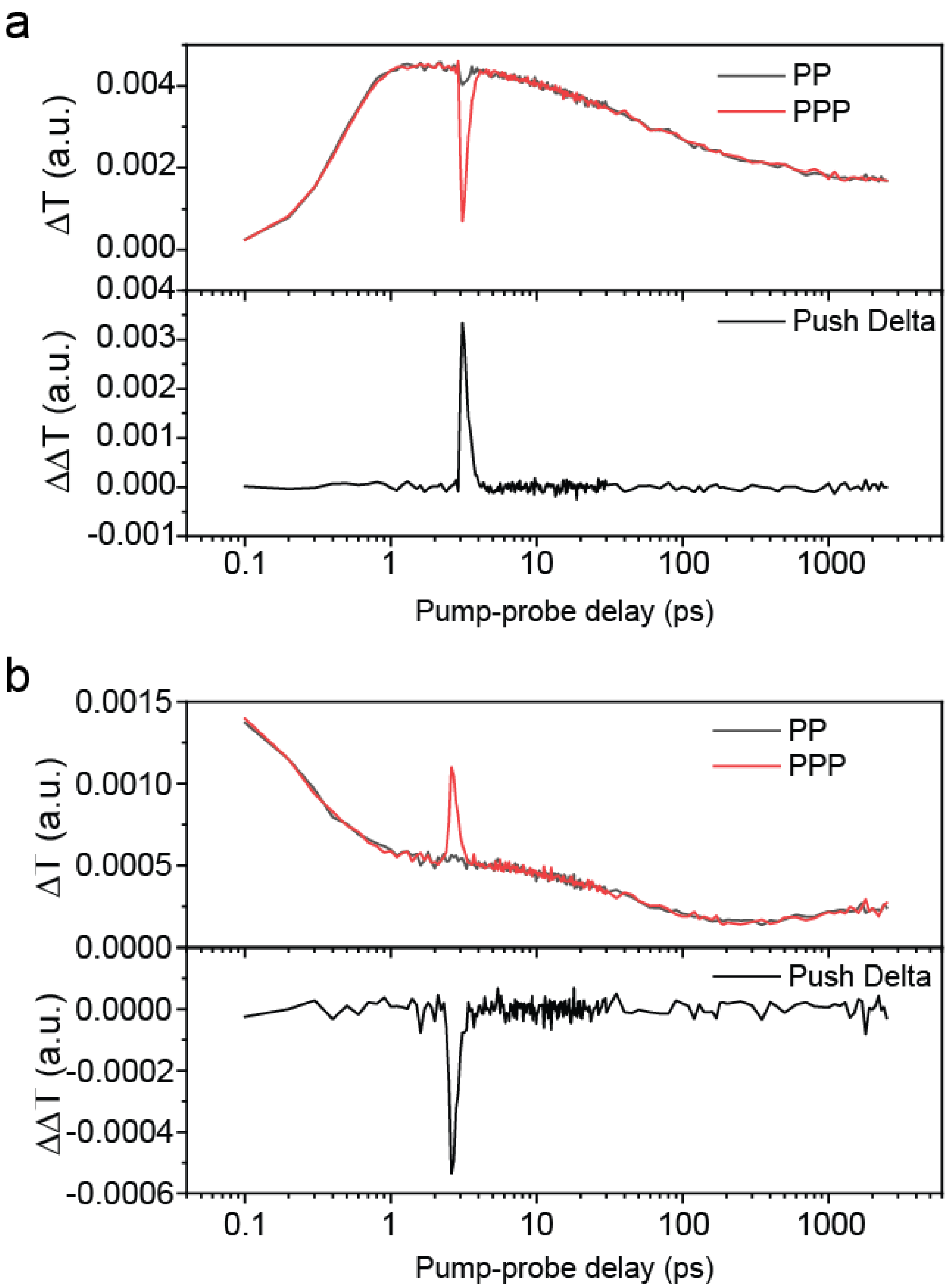

Supplementary Figure 22: **Raw pump-push-probe data for $MAPbBr_3$ nanocrystals.** Pump-push-probe kinetics for $MAPbBr_3$ NCs in anhydrous toluene solutions. The sample was pumped at 3.10 eV (40 μJ cm^{-2}), pushed at 1.03 eV (2.0 mJ cm^{-2}), and probed at 2.3 and 2.45 eV for the upper and lower panels, respectively. Light grey, red, and dark grey correspond to PP, PPP, and push delta, respectively.

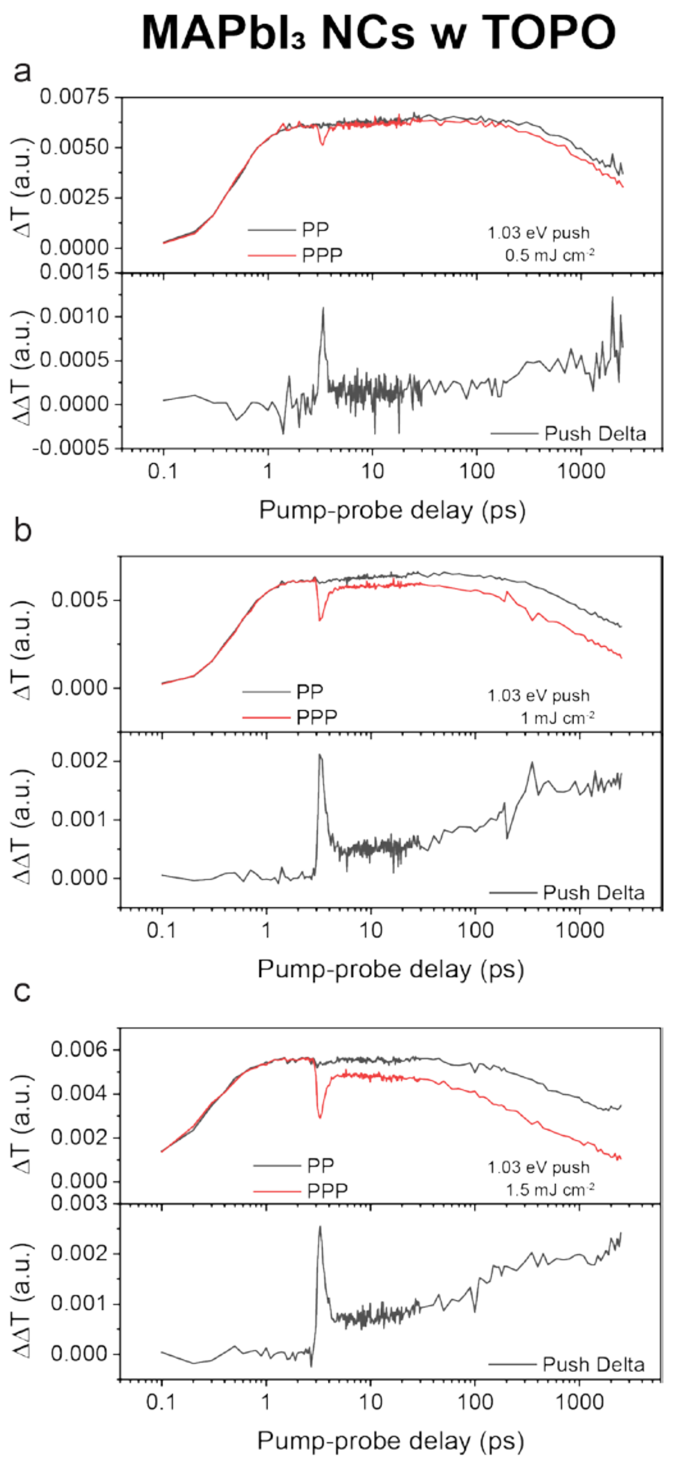

Supplementary Figure 23: **Raw pump-push-probe data for ligand exchanged $MAPbI_3$ nanocrystals.** Pump-probe (black), pump-push-probe (red) and differential pump-push-probe (grey) kinetics for TOPO ligand exchanged $MAPbI_3$ NCs in anhydrous toluene solutions. The samples were pumped at 2.07 eV (10 μJ cm^{-2}, $\langle N \rangle = 0.88$), probed at 1.7 eV, and with a push energy of 1.03 eV (0.5 to 1.5 mJ cm^{-2}). Light grey, red, and dark grey correspond to PP, PPP, and push delta, respectively.

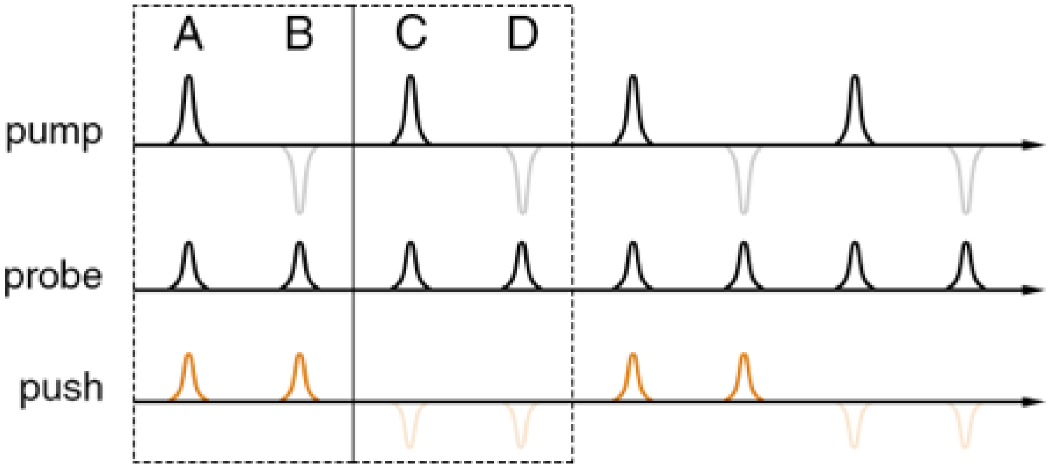

Supplementary Figure 24: **Schematic of the pulses sequence in the pump-push-probe experiment**. In our setup, the pump is modulated by a chopper, while a mechanical shutter modulates the push.

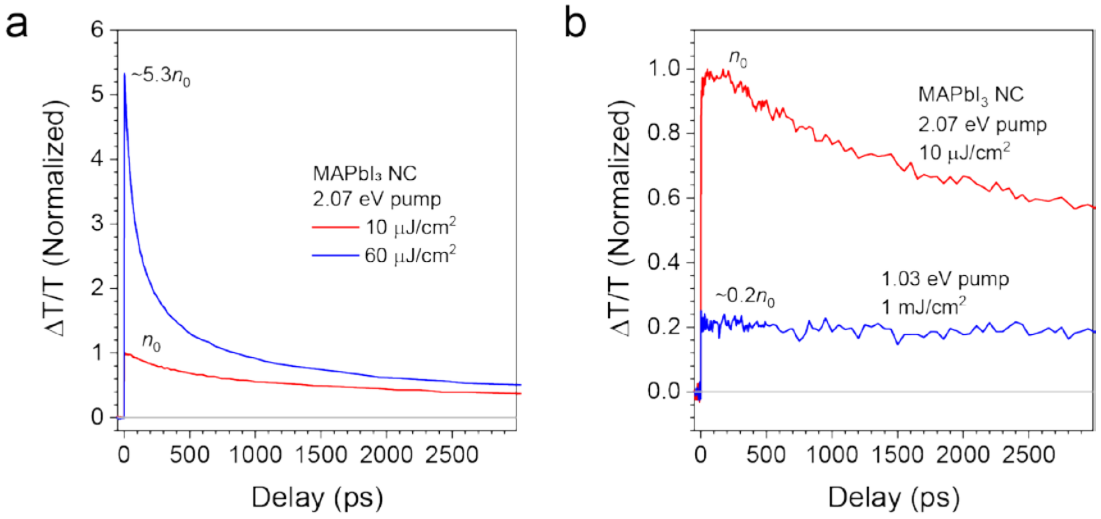

Supplementary Figure 25: Evaluation of push direct absorption. (a) Pump-probe kinetics at the band-edge (1.72 eV) of our $MAPbI_3$ colloidal NCs, pumped with 2.07 eV at 10 μJ cm^{-2} and 50 μJ cm^{-2}, respectively. Comparing the two populations, the visible push can generate up to 530% of carriers of those generated by the visible pump. (b)Transient absorption kinetics at the band-edge (1.72 eV) of our $MAPbI_3$ colloidal NCs, pumped with 2.07 eV (at 10 μJ cm^{-2}) and 1.03 eV (at 1 mJ cm^{-2}). Comparing the two populations, the IR pump generates about 20% of carriers of those generated by the visible pump.

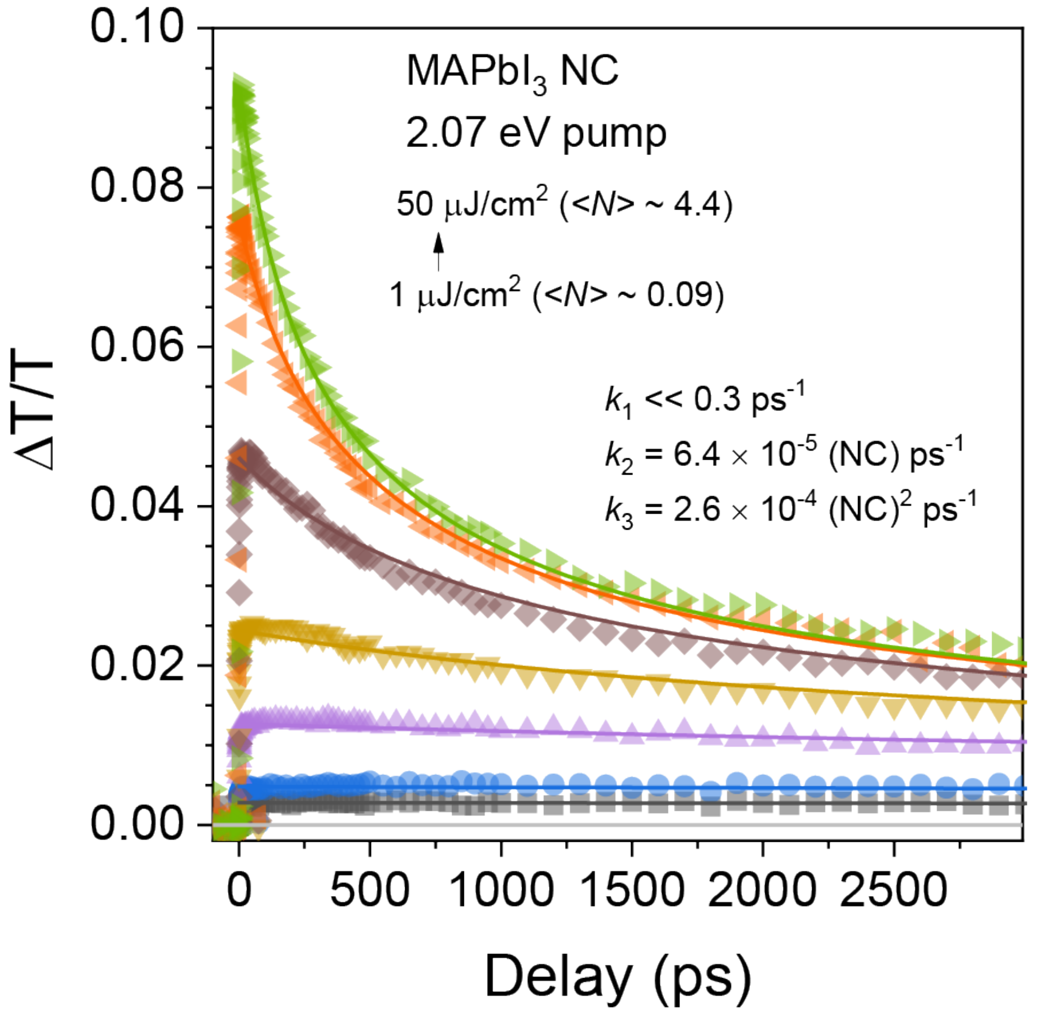

Supplementary Figure 26: Model fit of power dependent pump-probe measurements. Power dependent kinetics of $MAPbI_3$ colloidal NCs with 2.07 eV pump, for the determination of bimolecular and Auger recombination coefficient.

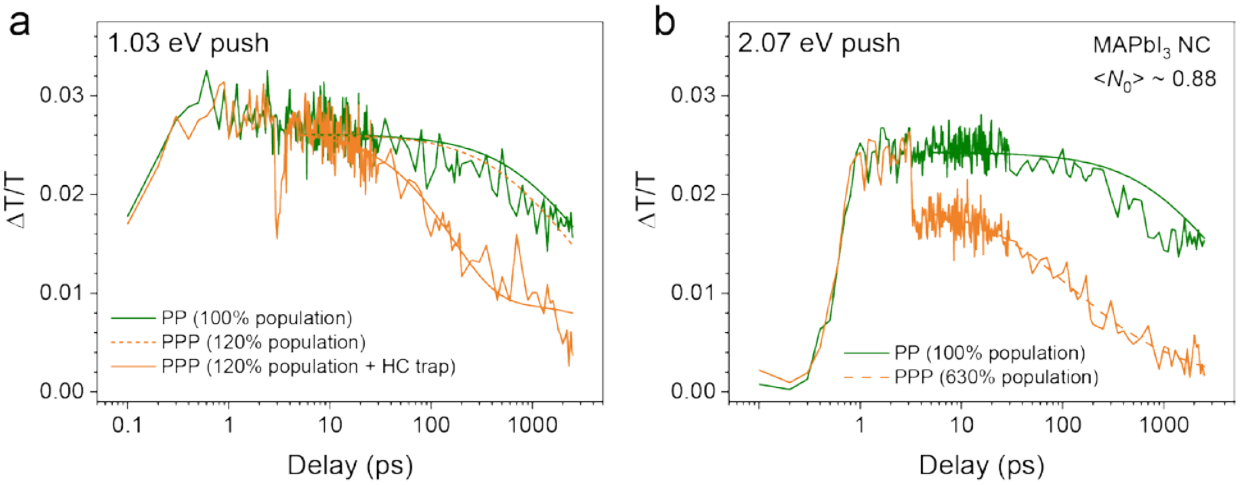

Supplementary Figure 27: Evaluation of Auger Artifacts and enhanced trapping. (a) Pump-probe (PP) and pump-push probe (PPP) kinetics of $MAPbI_3$ NCs reported in the manuscript (i.e. 2.07 eV pump at 10 μJ cm^{-2} and 1.03 eV push at 1 mJ cm^{-2}), together with the simulated post-push dynamics from the obtained bimolecular and Auger recombination coefficients. The solid orange line indicates the model with simulated HC traps. (b) Pump-probe (PP) and pump-push probe (PPP) kinetics of our sample reported in the manuscript (i.e. 2.07 eV pump at 10 μJ cm^{-2} and 2.07 eV push at 50 μJ cm^{-2}), together with the simulated post-push dynamics from the obtained bimolecular and Auger recombination coefficients.

Supplementary Table 1: **Fitting results of the hot carrier trapping model.**

	$MAPbI_3$	$MAPbI_3$ w/ TOPO	$MAPbBr_3$	$MAPbBr_3$ w/ TOPO
H_S (meV)	23 ± 1	22 ± 1	62 ± 4	34 ± 2
λ (meV)	800 ± 10	800 ± 10	770 ± 20	690 ± 20
k_2 (cm^3 s^{-1})	$(9.2 \pm 0.2) \times 10^{-8}$		-	
k_r (ns^{-1})	-		24 ± 2	

Supplementary Note 1 – Effects of IR/Visible Pushes Ground State Absorption

When performing PPP in the NIR-Visibile ranges, some artifacts may result from the direct ground state absorption of the push. To understand if this process could create artifacts in our results, we need to explain in detail our detection scheme for the PPP measurement. We refer to our recent publication[1], where the experimental setup was presented. As reported in Supplementary Figure 24, our measurements are based on lock-in detection, i.e., chopping the pump, which allows measuring the pump-probe signal. Thus, pump push probe measurements are obtained by introducing a third pulse utilizing a mechanical shutter. In this scheme, the PPP signal is obtained as the difference between the pump-probe signal with and without the push. Therefore, with reference to the Supplementary Figure 24, the signal we obtain is described as:

$$\Delta \mathrm{T}_{\text{push off}} = C - D$$
$$\Delta \mathrm{T}_{\text{push on}} = A - B$$
$$\Delta\Delta \mathrm{T} = \Delta \mathrm{T}_{\text{push off}} - \Delta \mathrm{T}_{\text{push on}}$$

Within this detection scheme, it is clear that the effect of the direct excitation from the push is canceled out when performing the $A - B$ operation. However, the presence of an additional population excited by the push and not directly detected by the lock-in detection could still influence the observed dynamics.

We performed two control "push-probe" experiments, to provide a quantification of the number of carriers excited from the ground state by our pump and push pulses during the reported pump-push-probe experiment. This push effect arises from the push absorption through linear absorption (visible push) and two-photon absorption (IR push) processes. As shown in Supplementary Figure 25a, using two different pump fluences: 2.07 eV (at 10 μJ cm^{-2}) and 2.07 eV (at 50 μJ cm^{-2}), we observe that the visible push can excite up to 530% of the original population excited by the pump. On the other hand, as shown in Supplementary Figure 25b, we performed TA measurement using two different pump energies: 2.07 eV (at 10 μJ cm^{-2}) and 1.03 eV (at 1 mJ cm^{-2}) to estimate the

effect of the IR push. Through two-photon absorption, the IR push can excite up to 20% of the original population in the PPP experiment. It is worth to stress that these effects are not observed in terms of ΔΔT values during the push (e.g., the presence of a negative ΔΔT due to increased excited state population), since our pump-push-probe technique is insensitive to this ground-state excitation by the push. However, the presence of an additional population could have an effect on the kinetics after the push.

To demonstrate in a quantitative way the possible effect of the push-induced population on the PPP kinetics, we performed power-dependent pump-probe to estimate the bimolecular and Auger relaxation coefficient in our $MAPbI_3$ colloidal nanocrystals (NCs). The population dynamics in the NCs can be simply modeled by the following rate equation:

$$\frac{\mathrm{d}}{\mathrm{d}t}\langle N\rangle = -k_1\langle N\rangle - k_2\langle N\rangle^2 - k_3\langle N\rangle^3 \tag{1}$$

where $\langle N\rangle$ is the average population of carriers per NC; k_1 is the trap-assisted recombination rate, k_2 bimolecular recombination coefficient; and k_3 is the Auger recombination coefficient. By global fitting this equation to our power-dependent kinetics with k_1, k_2 and k_3 as the shared parameters, we could obtain values for these coefficients, i.e., k_2 = 6.4 × 10^{-5} (NC) ps^{-1} and k_3 = 6.4 × 10^{-5} $(NC)^2$ ps^{-1}. Meanwhile, the maximum range of our delay time measurement is too short for an accurate determination of k_1. The result is shown in Supplementary Figure 26. By using these values, we could simulate the corresponding carrier dynamics after-push due to the additional carriers from the push ground-state absorption. In the case of IR push (Supplementary Figure 27a), it is worth to note that the additional carriers indeed cause slightly faster decay dynamics, but the change is well within the experimental noise. Therefore, we conclude that the Auger effect caused by 2PA of the IR push cannot account for the considerable lifetime variation observed. A different kinetics model based on our original interpretation of hot carrier (HC) trapping is therefore needed to describe fully the fast decay dynamics in the post-push dynamics. Herein, after the initial push

fast relaxation (i.e., t longer than 5 ps), we postulate that a fraction of the carriers was ported over to the trap potential and undergoes an exponential decay via the trap-assisted recombination with a rate of k_{tr} = 0.01 ps^{-1} (as described in the Main Text); while the remaining fraction stays at the free-carrier potential and undergoes decay via the normal k_1, k_2, and k_3 rates. The resultant dynamics is shown as the solid organce line in Supplementary Figure 27a, where it successfully reproduces the experimental results. On the other hand, the visible push generates a larger additional population and therefore results in more marked changes in the kinetics, as reported in Supplementary Figure 27b. The simulated dynamics (that consider an additional 530% of push induced population) perfectly reproduces the experimental data. Therefore, differently from what we observed for the IR push, we can assign the faster recombination to the enhanced bimolecular and Auger recombinations caused by the visible push absorption from the ground state.

Supplementary Note 2 -- Hot-carrier trapping model

We described the hot-carrier trapping dynamics in the framework of the Marcus theory of charge transfer from free carriers to trapped carriers potential energy surfaces. We described population dynamics in both free carriers (N) and shallow-trapped carriers (N_T) states as the following:

$$\frac{dN(t)}{dt} = -k_2 N^2(t) - \int_0^\infty k_T(\epsilon)\, n(\epsilon,t)\, \mathrm{d}\epsilon + e^{\Delta G_0/k_B T} \int_0^\infty k_T(\epsilon)\, n_T(\epsilon,t)\, \mathrm{d}\epsilon, \quad (2)$$

$$\frac{dN_T(t)}{dt} = -k_{tr} N_T(t) + \int_0^\infty k_T(\epsilon)\, n(\epsilon,t)\, \mathrm{d}\epsilon - e^{\Delta G_0/k_B T} \int_0^\infty k_T(\epsilon)\, n_T(\epsilon,t)\, \mathrm{d}\epsilon. \quad (3)$$

Here, the independent variable ϵ is the carrier energy axis; k_2 and k_{tr} are the radiative bimolecular coefficient and non-radiative recombination rate from the free-carrier and trapped-carrier potentials, respectively; $n(\epsilon,t)$ and $n_T(\epsilon,t)$ are the thermally equilibrium carrier distributions of the free-carrier and trapped-carrier potentials, with carrier temperature $T(t)$ and 300 K, respectively; $k_T(\epsilon)$ is

the population exchange rate as a function of ϵ between the two potentials; and ΔG_0 is the Gibbs free energy. The electron and hole dynamics is assumed to be symmetrical. Another assumption is that the radiative recombination process happens in the bulk of the NCs. Note that for $MAPbBr_3$ NCs, the first term presented in Supplementary Equation 2 was modified to account for the more excitonic nature of the recombination of these samples. Here, the radiative bimolecular recombination term is replaced by a monomolecular radiative recombination term with rate k_r [i.e., $-k_2N^2(t) \to -k_rN(t)$].

The population exchange rate between the two potentials is described in Marcus theory and is related to the reorganization energy, λ, and carrier-trap electronic coupling energy, H_S, by:

$$k_T(\epsilon) = \frac{2\pi}{\hbar}|H_S|^2 \frac{1}{\sqrt{4\pi\lambda k_B T_L}} e^{-(\lambda+\epsilon+\Delta G^0)^2/4\lambda k_B T_L} \quad (4)$$

where T_L is the lattice temperature, which was assumed to be 300 K (room temperature). We assume the phenomenological carrier temperature to be $T(t) = 300\ \mathrm{K} + T_0 \exp(-t/\tau_c)$, where $T_0 = \delta_E/k_B$ is the excess carrier temperature photoexcited with an excess energy (δ_E) that cools down with cooling time constant (τ_c) is set to be 0.4 ps. We also assume that the carrier distribution is governed by the Boltzmann distribution, which is given by:

$$f(\epsilon, N, T) = \exp\left\{-\frac{\epsilon - \mu(N,T)}{k_B T}\right\}. \quad (5)$$

In this framework, given the density of states of a parabolic band $g(\epsilon) = (1/2\pi^2)(2m^*/\hbar^2)^{3/2}\epsilon^{1/2}$, the chemical potential is given by:

$$\mu(N,T) = k_B T \ln\left[\frac{2N}{\pi}\left(\frac{\pi\hbar^2}{2m^* k_B T}\right)^{\frac{3}{2}}\right]. \quad (6)$$

The m^* refers to the effective mass of the carriers. These parameters are related to the total number of carriers by the following relation:

$$N = \int_0^{\infty} n(\epsilon)\mathrm{d}\epsilon = \int_0^{\infty} g(\epsilon)f(\epsilon)\mathrm{d}\epsilon. \quad (7)$$

Solving Supplementary Equation 2 and 3, the temporal dynamics of the populations can be obtained. The PLQY of the system with initial photoexcited carrier population $N(0) = N_0$ (set to be 10^{16} cm^{-3}) can therefore be calculated as:

$$\mathrm{PLQY} = \frac{k_2 \int_0^{\infty} N^2(t)\mathrm{d}t}{N_0} \quad \text{for iodide system, and} \quad (8a)$$

$$\mathrm{PLQY} = \frac{k_{\mathrm{r}} \int_0^{\infty} N(t)\mathrm{d}t}{N_0} \quad \text{for bromide system.} \quad (8b)$$

Based on our pump-push-probe experimental results, we set the $\Delta G_0 = 0$ (seen at the sample probe energy); with $k_{\mathrm{tr}} = 0.01$ ps^{-1}. The H_S, λ, and k_2 (or k_r for bromide NCs) are set as fitting parameters. The results of the fitting for both $MAPbBr_3$ and $MAPbI_3$ NCs are shown in Supplementary Table 1, as can be seen in Figure 3d-e in Main Text, the model successfully reproduced the experimental results. Our result also implies that the TOPO improves the quantum yield by reducing the carrier-trap electronic coupling energy (H_S), without significantly changing the reorganization energy (λ).

Supplementary References

1 Lim, S. S. *et al.* Hot carrier extraction in $CH_3NH_3PbI_3$ unveiled by pump-push-probe spectroscopy. *Science Advances* **5**, eaax3620 (2019).
